# Drug Therapy Considerations in Arrhythmias in Children

**Published:** 2008-08-01

**Authors:** V Ramesh Iyer

**Affiliations:** Assistant Professor of Pediatrics, University of Connecticut School of Medicine, Division of Cardiology, Connecticut Children's Medical Center, Hartford, CT 06106, U.S.A

**Keywords:** Arrhythmia, Anti-arrhythmic drugs, Supra ventricular tachycardia, ventricular tachycardia

## Abstract

The understanding of the mechanisms of action and side effects of different antiarrhythmic drugs (AAD) are essential for their appropriate use. With the advent of effective and safe ablation therapy, AAD therapy has undergone dramatic changes. This review attempts to understand the rationale behind the clinical use of medical management of different arrhythmias.

## Introduction

With the market share of drug use in Pediatric population being relatively small, drug companies have little incentive for drug trials. The use of AADs in children is often off label and derived from the adult experience. The efficacy of arrhythmia control in children may vary considerably. It is important to understand the following definitions:

***Pharmacokinetics:*** It is the study of drug concentrations after oral or intravenous (IV) administration

***Pharmacodynamics:*** It is the study of the variability in the response to the drug concentrations.

***Pharmacogenetics:*** It is the study of inherited variability in response to drug therapy.

***Bioavailabilty:*** It is the amount of drug reaching the systemic circulation usually after oral administration as compared to IV therapy. Bioavailabilty depends on 1) Drug absorption 2) Metabolism before reaching systemic circulation (such as hepatic or enteral metabolism).

***Steady state:*** State at which the amount of drug leaving the plasma and tissues equals the amount entering the plasma and tissues after chronic drug administration.

***Elimination half life:*** Time required for the plasma concentration to fall by 50%. In 2 half lives 75% of the drug is eliminated, in 3 half lives 87.5% and almost complete elimination in 5-6 half lives.

Drug elimination is defined as a "first order" process if the rate at which the drug is eliminated depends on the plasma concentration. A "zero order" process is when the drug is eliminated per unit time regardless of the drug concentration e.g. ethanol.

***Clearance:*** It is the amount of drug cleared by metabolism per unit time. Factors that affect clearance include organ dysfunction and concomitant drug therapy that affect metabolism. Rapid clearance of drugs such as esmelol reflects activity of plasma esterases [1] whereas the clearance of adenosine is due to widespread drug uptake into intracellular sites (RBCs) [2].

***Volume of distribution:*** The central volume of distribution is the volume into which the IV bolus distributes. Dose adjustment may be required in situations of altered volume of distribution such as congestive heart failure. The half life of a drug is directly proportional to the volume of distribution and inversely proportional to the clearance.

Drug clearance may be reduced by 1) Dysfunction of the eliminating organ, 2) Concomitant drug therapy that inhibits metabolic or transport pathway, 3) Defective function of drug eliminating protein.

It is a common misconception that drug need to administered once per half life. Optimum dosing is determined not only by drug's elimination rate but also by the margin between the minimally effective and maximally tolerated plasma concentration.

## Classification and mechanism of action Antiarrhythmic Drugs

The modified Singh-Vaughan Williams classification [3] is as follows:

### Class I agents

#### Class I A

These reduce the conduction velocity and prolong repolarization thus prolonging action potential duration. These include Quinidine, Procainamide and Disopyramide

*Quinidine:* It is derived from the bark of cinchona tree and delays depolarization and repolarization by Na channel [4] and IKi channel blocking effects respectively. It can cross placenta and gets excreted in breast milk. It is effective in atrial arrhythmias such as atrial flutter and fibrillation, re-entry SVTs and ventricular arrhythmias such as VT. Use of quinidine has declined because of potent side effects such as diarrhea, abdominal cramping, thrombocytopenia, hemolytic anemia, decreased hearing and tinnitus, QRS widening and proarrhythmia [5]. Dosage used is 30-60mg/kg/day in four divided doses.

*Procainamide:* It prolongs action potential duration (APD) by sodium channel blocking effect. It is metabolized to N-acetyl procainamide (NAPA) that has class III effects. Some additional effects include alpha adrenergic blocking (decreased sympathetic efferent activity) that may cause hypotension and vagolytic action that may increase the AV node response of atrial arrhythmias such as atrial flutter and fibrillation. It crosses placenta and is excreted in breast milk.  It is effective in a wide range of atrial and ventricular arrhythmias. The intravenous form is often used as first line of therapy in postoperative (after CHD repair) atrial and ventricular arrhythmias. Side effects include hypotension, GI side effects, drug induced lupus [6], hemolytic anemia and rarely proarrhythmia. Oral dosing is 40-100 mg/kg/day in 4-6 divided doses. IV loading is done with 5-15mg/kg followed by 40-100 mcg/kg/min. Procainamide/NAPA levels are helpful in monitoring therapy especially in slow acetylators (10-40% of the population)

*Disopyramide:* This has similar action as quinidine without the adrenergic effects. It is effective in refractory vasodepressor syncope [7]. The negative inotropic effect inhibits the ventricle from becoming hypercontractile which is the trigger for the vasodepressor reflex. The dose used is 20-30 mg/kg/day in 3-4 divided doses. Side effects include anticholinergic effects such as urinary retention, constipation blurred vision and dry mouth. Renal clearance may be affected by betablockers. 

#### Class I B

These drugs shorten APD and repolarization by bocking the fast Na channel activity and include lidocaine, mexilitine, phenytoin, tocainide and moricizine.

*Lidocaine:* Most of the effects are below the His bundle and results in overall homogeneity of the ventricular muscle repolarization. It has an extensive first pass hepatic metabolism with a rapid distribution phase. It has poor oral absorption and it does cross the placenta. It is effective in acute management of VT especially torsades (1). IV dosing is 1-4 mg/kg bolus followed by infusions of 20-50mcg/kg/min. Side effects include CNS symptoms such as numbness, altered sensorium, seizures and apnea.

*Mexilitine:* This is an oral form of lidocaine with similar Na channel effects. It is rapidly absorbed from the GI tract with extensive hepatic metabolism. It is effective in chronic therapy of some VTs and also LQTS (type III) [8]. It is known to cross the placenta and has been used on some case of fetal ventricular arrhythmias from congenital prolongation of the QT interval. Dosing range is 2-8 mg/kg/day in 3 divided doses. Side effects include skin rash and GI intolerance.

*Phenytoin:* It has similar effects as Lidocaine on the Na channel but also has Ca channel blocking and sinus node and AV node effects in higher concentrations. Its effect of depressing phase 4 depolarization is used in treatment of digoxin toxicity. It is used in some forms of refractory ventricular arrhythmias. It crosses the placental barrier and is know to have a potent fetal teratogenic effect. Maintenance dosing is 3-6 mg/kg day in 2 divided doses after an initial loading of 10-15 mg/kg. Side effects include hypotension, nystagmus, ataxia, gingival hyperplasia, skin rash (may progress to Steven Johnson's syndrome) and fetal hydantoin syndrome (cleft lip/palate, microcephaly, mental retardation, finger/nail hypoplasia and CHD) if used in pregnancy.

#### Class I C

These agents are potent in blocking Na and affecting repolarization. They also have an effect on inward K channel (iK) and the slow inward Ca (iCa) current. They include flecanide, propafenone and encanide.

*Flecanide:* It prolongs ventricular APD and refractory periods leading to QRS prolongation. In the specialized conduction tissue, it shortens the refractory period and decreases automaticity. It crosses the placenta effectively (upto 70% of maternal levels) and can be used in fetal arrhythmias. In the pediatric population, it has been used in a wide variety of arrhythmias including re-entry SVT, EAT, IART and some forms of VTs [9]. Its use in the presence structural heart disease (ischemic heart disease and CHD) has been questioned by CAST study [10].  Dosing is 80-180mg/m^2^/day in 2-3 divided doses. Flecanide levels can be used to monitor therapy and toxicity. Side effects include blurry vision, prolongation of the QRS duration and proarrhythmia especially in the presence of structural heart disease.

*Propafenone:* Effects are similar to flecanide and also has mild betablocking effects, and some effects on the slow inward Ca current and delayed outward potassium current. It is effective in especially automatic atrial tachycardia [11]. It is extensively metabolized in liver. Oral dosing is 150-500 mg/m^2^ in 3-4 divided doses. Side effects include blurry vision, lupus like syndrome and conduction system disturbances including bundle branch blocks.

### Class II drugs

These are agents with beta adrenergic blocking properties. These include beta-1 (cardiac) selective (atenelol, metoprolol), non selective (propranolol, nadalol) and drugs that have intrinsic sympathomimetic activity (ISA) (Pindalol)

*Propranolol:* It is the most commonly used betablocker which has membrane effects on the Na channel and weak Ca channel effects with higher doses. It has little effect on the APD but prolongs intranodal conduction (increases AH interval and Wenckebach block). Hepatic metabolism and clearance is extensive. It crosses the placenta easily and fetal hypoglycemia is a concern with maternal use. It is widely used as first choice in re-entry SVT of all ages (including newborns), some ventricular arrhythmias (especially catecholamine sensitive) and is also the drug of choice in LQTS (2). It blunts the imbalance between the sympathetic and parasympathetic influence and hence heterogeneity of the ventricular muscle refractoriness in post MI patients and hence improves survival. Dosing is between 1-4 mg/kg per day in 3-4 divided doses. Co-administration with Ca channel blockers may cause synergistic negative inotropic effect on contractility. Side effects include bradycardia, hypotension [12], fatigue, depression and may exacerbate bronchospasm.

*Atenelol:* It is another commonly used long acting betablocker that has selective beta-1 effects and does not cross the blood brain barrier. It is used in re-entry SVTs and ventricular tachycardia [13] in older children with convenience of once or twice a day dosing. Oral dosing is 1-2 mg/kg per day. Side effects are similar to propranolol but less common.

*Nadalol:* It is a non selective betablocker with similar actions as propranolol but has a longer half life. It is used in reentry SVT [14] and autonomic syndromes (vasodepressor syncope). Oral dosing is 1-2 mg/kg per day in 1-2 divided doses.

*Esmolol:* It is an IV form of cardioselective betablocker that has half life of only 7-10 mins. The predominant sites of action are the sinus and AV nodes. It is used in the acute treatment of re-entry SVTs and catecholamine sensitive VTs [15] and also rate control of non-reentry tachycardia such as atrial flutter and EAT. IV dosing is 500 mcg/kg bolus over 1-2 mins followed by an infusion of 50-200 mcg/kg/min. Side effects include hypotension and bradycardia.

Other betablockers that have not been used frequently in children include Pindalol and Carvedilol. Pindolol has potent ISA and may be used in arrhythmia control in patients with sinus or AV node disease as it doesn't cause much bradycardia. Carvedilol has additional vasodilator and antioxidant properties and improves ventricular function which has made it use popular in chronic CHF.

### Class III drugs

These agents effect the membrane repolarization and thus prolong the APD and refractory period. The drugs have predominant K channel effects with some Na and Ca channel blocking properties. Higher doses also have beta and alpha adrenergic blockade contributing to its therapeutic actions. These result in reduction in membrane excitability of all myocardial tissue. Amiodarone, Sotalol, Ibutalide and dofetalide belong to this class.

*Amiodarone:* It is considered to be one of the most effective antiarrhythmic agents even in the presence of CHD and heart failure. It is effective in a variety of reentry SVTs, atrial flutter and fibrillation, automatic EATs and life threatening ventricular arrhythmias [16]. Oral dosing is 10-15 mg/kg as loading over a period of 5-10 days followed 2-5 mg/kg as once a day dose. IV dosing is safe [17] and is recommended in a 1-5 mg/kg/dose over a period of 1-2 hours slowly followed by a maintenance infusion of 5-15 mcg/kg/min. Higher loading doses may be required in some cases. Side effects may be significant especially with long term administration and include hypothyroidism, skin photosensitivity, chemical hepatitis, pulmonary fibrosis and corneal microdeposits that are reversible. Regular thyroid function, liver function and pulmonary function tests are essential to avoid undesirable effects and may need reducing the dose or supplemental thyroxine. Cardiac side effects include sinus and AV node dysfunction and rarely proarrhythmia (such as torsades).

*Sotalol:* It is a non-selective betablocker with all above mentioned class III effects. Its use in postoperative chronic management of several arrhythmias such as atrial flutter, EAT and VT is well described. Oral absorption is good and transplacental passage of the drug has been used in treating fetal arrhythmias. Oral dosing is 90-200 mg/m^2^ in 3 divided doses. Side effects include bradycardia, hypotension and prolongation of QTc with a risk of torsades [18].

### Class IV drugs

These bock the Ca channels and have a predominant effect on the sinus and AV node.

*Verapamil:* It is effective in depressing enhanced automaticity and is a potent alpha adrenergic blocker. The Ca channel blocking properties are more prominent at faster heart rates. It has a large volume of distribution and is metabolized in the liver. It is mainly used in re-entry SVTs, hypertrophic cardiomyopathy and some left sided VTs. Oral dosing is between 4-15mg/kg/day in 3-4 divided doses. Sustained release preparations are available and are administered once or twice daily. Side effects include headaches, rashes, constipation, bradycardia and postural hypotension. Use of the IV preparation in infants can result in asystole and cardiovascular collapse [19] and hence is contraindicated.

*Diltiazem:* It blocks the inward Ca channel activity and has predominant effect on the sinus and AV node. It has been used predominantly in control of hypertension and acute treatment of SVT [20]. Its use is similar to verapamil and dosing recommendations are 0.5-2 mg/kg/day in 2-3 divided doses. Side effects include bradycardia and postural hypotension.

### Other agents

*Adenosine:* It is an endogenous purigenic agent that increases K channel conductance and depresses inward Ca current resulting in transient AV block. It has wide therapeutic and diagnostic use in emergency room management of re-entry (3) and automatic SVTs such as ectopic atrial tachycardia (4) and some forms of adenosine sensitive VTs. IV dosing is 150-300 mcg/kg given rapidly because of its short half life (few seconds) [21]. Side effects include flushing, hypotension, chest pain (transient coronary spasm), bronchospasm and atrial fibrillation.

*Digoxin:* This has several mechanisms of action. The direct effects include binding to Na-K ATPase complex and thus inhibiting Na channel. The inotropic response is due to Ca loading from higher intracellular Na concentration from the enhancement of Na-Ca pump. It also increases parasympathetic output [22] and inhibits norepinephrine release. The drug is excreted in the urine unchanged and also crosses the placenta (hence its use as drug of choice in fetal arrhythmias).  Oral dosing is 30-50 mcg/kg loading over 24 hrs in divided doses followed by 7-10 mcg/kg per day. IV dosing is two-thirds of oral dosing. Side effects include nausea, GI and visual disturbance. Cardiovascular effects of digoxin toxicity include bradycardia, AV block and ventricular arrhythmias. Electrolyte and renal abnormalities (especially hypokalemia and hypomagnesemia) potentiate digoxin toxicity.

## Conclusion

In summary, antiarrhythmic drug therapy in children is very effective in situations where ablation is not preferred. Side-effects of different drugs and combinations need to be taken into consideration and monitored during treatment.

## Figures and Tables

**Figure 1:**
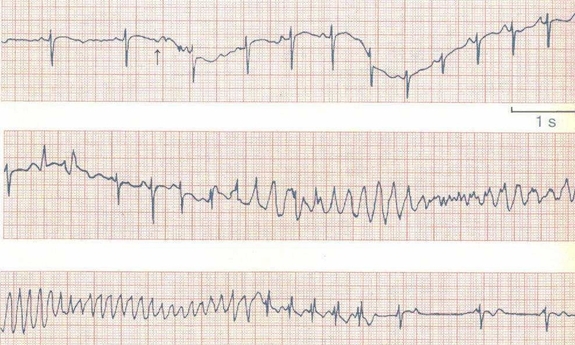
EKG of a 6 yr old with recurrent episodes of syncope showing torsades who successfully treated with IV Lidocaine

**Figure 2:**
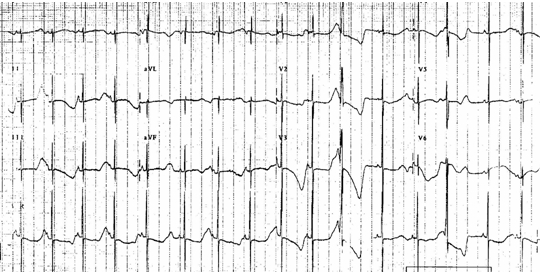
EKG of a 2 month old girl with LQTS (QTC of 520ms.) who was treated with propranolol.

**Figure 3:**
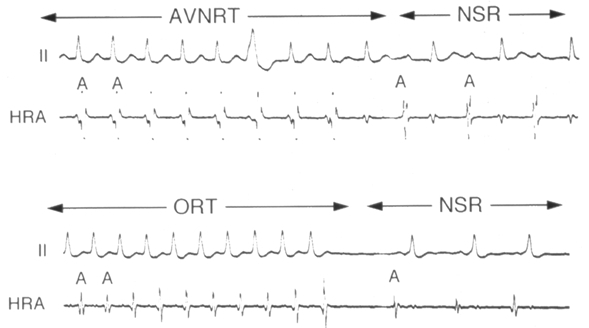
Effect of Adenosine on re-entry SVT. (Lead II and HRA tracings) Upper panel shows response in AVNRT. Termination of tachycardia is seen after a ventricular response. Lower panel shows the response in ORT where the tachycardia terminates after an atrial response. (AVNRT- AV node re-entry tachycardia; ORT-orthodromic re-entry SVT, NSR- normal sinus rhythm, HRA- high right atrium).

**Figure 4:**
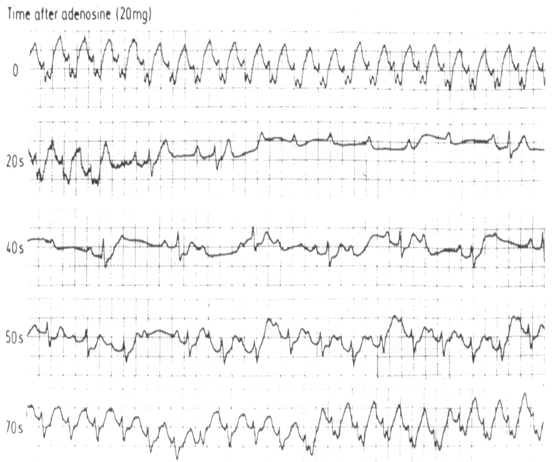
Effect of Adenosine in Ectopic atrial tachycardia. With blocking of the AV node the ventricular response is slowed but tachycardia continues. Response lasts less than 30 seconds.
